# Web-based tools applied to therapy and counseling during the COVID-19 pandemic

**DOI:** 10.1192/j.eurpsy.2021.786

**Published:** 2021-08-13

**Authors:** A.R. Dores, A. Geraldo, I. Carvalho, F. Barbosa

**Affiliations:** 1 Laboratory Of Neuropsychophysiology (fpceup); Center For Rehabilitation Research (ess-p.porto), Faculty of Psychology and Education Sciences; School of Health, Polytechnic of Porto, Porto, Portugal; 2 Laboratory Of Neuropsychophysiology, Faculty of Psychology and Education Sciences, Porto, Portugal; 3 Clinical Neurosciences And Mental Health And Cintesis, School of Medicine, University of Porto,, Porto, Portugal

**Keywords:** Web-based tools, Therapy, e-health, COVID-19

## Abstract

**Introduction:**

Web-based tools allowed the provision of psychological counseling and therapy at-distance during the COVID-19 lockdown. Nevertheless, psychologists’ attitudes towards the adoption of these tools and its impact in their professional practices need to be further explored.

**Objectives:**

The objective was to study the use of web-based tools on psychological practices before and during COVID-19 lockdown, trying to identify changes on psychologists’ professional practices related to the pandemic, as well as to explore factors that could have affected such changes.

**Methods:**

One-hundred and eight psychologists filled-in an online survey, developed for the purposes of this study, during mandatory lockdown. The study was disseminated by mailing list, social networks, and by the Portuguese Psychologists Association.

**Results:**

The results have shown that psychologists kept providing their services during lockdown thanks to the adoption of web-based tools. Although psychologists have recognized that additional precautions were needed for at-distance practice in comparison to in-person interventions, the experience of using IC technologies in clinical practice was described as positive, ensuring clients’ adherence with positive results. Additionally, despite psychological services were maintained on a larger scale by psychologists with more years of experience, professionals with average experience stated more favorable attitudes towards the use of web-based tools in counseling and therapy.

**Conclusions:**

Although the implementation of ICT based practice was enforced by current circumstances, the experience that psychologists gathered and shared during the lockdown can guide future professional practice, improving and fostering the replication of best practices at distance.

